# Networks Are Associated With Acupuncture Treatment in Patients With Diarrhea-Predominant Irritable Bowel Syndrome: A Resting-State Imaging Study

**DOI:** 10.3389/fnhum.2021.736512

**Published:** 2021-10-14

**Authors:** Tingting Zhao, Lixia Pei, Houxu Ning, Jing Guo, Yafang Song, Junling Zhou, Lu Chen, Jianhua Sun, Zhongping Mi

**Affiliations:** ^1^Department of Acupuncture-Moxibustion and Rehabilitation, Affiliated Hospital of Nanjing University of Chinese Medicine, Nanjing, China; ^2^Acupuncture and Moxibustion Disease Project Group of China Evidence-Based Medicine Center of Traditional Chinese Medicine, Nanjing, China; ^3^Department of Traditional Chinese Medicine, The Affiliated Hospital of Nanjing University Medical School, Nanjing, China; ^4^College of Acupuncture, Massage, Health and Rehabilitation, Nanjing University of Chinese Medicine, Nanjing, China

**Keywords:** irritable bowel syndrome, independent component analysis, fMRI, acupuncture, functional connectivity, mechanism

## Abstract

**Background:** Irritable Bowel Syndrome (IBS), as a functional gastrointestinal disorder, is characterized by abdominal pain and distension. Recent studies have shown that acupuncture treatment improves symptoms of diarrhea-predominant irritable bowel syndrome (IBS-D) by altering networks in certain brain regions. However, few studies have used resting-state functional magnetic resonance imaging (fMRI) to compare altered resting-state inter-network functional connectivity in IBS-D patients before and after acupuncture treatment.

**Objective:** To analyze altered resting-state inter-network functional connectivity in IBS-D patients before and after acupuncture treatment.

**Methods:** A total of 74 patients with IBS-D and 31 healthy controls (HCs) were recruited for this study. fMRI examination was performed in patients with IBS-D before and after acupuncture treatment, but only at baseline in HCs. Data on the left frontoparietal network (LFPN), default mode network (DMN), salience network (SN), ventral attention network (VAN), auditory network (AN), visual network (VN), sensorimotor network (SMN), dorsal attention network (DAN), and right frontoparietal network (RFPN) were subjected to independent component analysis (ICA). The functional connectivity values of inter-network were explored.

**Results:** Acupuncture decreased irritable bowel syndrome symptom severity score (IBS-SSS) and Hamilton Anxiety Scale (HAMA). It also ameliorated symptoms related to IBS-D. Notably, functional connectivity between AN and VAN, SMN and DMN, RFPN and VAN in IBS-D patients after acupuncture treatment was different from that in HCs. Furthermore, there were differences in functional connectivity between DMN and DAN, DAN and LFPN, DMN and VAN before and after acupuncture treatment. The inter-network changes in DMN-VAN were positively correlated with changes in HAMA, life influence degree, and IBS-SSS in IBS-D.

**Conclusion:** Altered inter-network functional connectivity is involved in several important hubs in large-scale networks. These networks are altered by acupuncture stimulation in patients with IBS-D.

## Introduction

Irritable bowel syndrome (IBS) is one of the most common functional gastrointestinal disorders characterized by abdominal pain, distension, and changes in stool frequency and form (Lacy et al., 2016; Ford et al., 2020). It is estimated that IBS affects approximately 11% of the global population (Canavan et al., 2014). In South-East China, the prevalence of IBS ranges between 5.9 and 7% (Long et al., 2017). The prevalence is 12% in northern Europe (Lovell and Ford, 2012) and 7–16% in the United States (Camilleri, 2021). IBS can be categorized into four subgroups: constipation-predominant IBS, diarrhea-predominant IBS (IBS-D), mixed IBS, and unclassified IBS (Lacy et al., 2016). IBS-D is the most common subtype accounting for 40% of all IBS patients (Lovell and Ford, 2012). The precise pathophysiology of IBS remains poorly elucidated. It is generally viewed as a dysfunction of the brain-gut axis (Tsang et al., 2016). Studies report that IBS negatively affects the quality of life and imposes high economic burden on the social and family (DiBonaventura et al., 2011; Doshi et al., 2014). The current treatments for IBS include symptomatic treatments, such as non-pharmacological (e.g., dietary and lifestyle modifications, psychological therapy), and pharmacologic therapies (Łodyga et al., 2012; Cangemi and Lacy, 2019). However, pharmacologic therapies in IBS are associated with serious side effects, such as headache, dizziness, dry mouth, and insomnia (Nam et al., 2018; Niewinna et al., 2020).

Acupuncture, a traditional Chinese medicine therapy, has been extensively used for the treatment of gastrointestinal disorders, and it may also be a promising complementary and alternative therapy for IBS (Manheimer et al., 2012; Guo et al., 2020; Zhang et al., 2020; Chen Y. et al., 2021). A series of clinical studies have shown that acupuncture can alleviate IBS-related symptoms, including abdominal pain, abdominal distension, bowel movement, scores for anxiety, depression, and sleep quality (Li et al., 2017; Pei et al., 2020; Guo et al., 2021; Sun et al., 2021). Although acupuncture can effectively control IBS symptoms, the underlying regulatory mechanism is elusive. Studies have explored involvement of the brain-gut axis as a mechanism mediating the benefits of acupuncture on IBS (Song et al., 2020; Chen L. et al., 2021; Yaklai et al., 2021).

Resting-state functional magnetic resonance imaging (fMRI) is an effective, non-invasive, and novel approach used to explore brain function mechanisms that does not require patients to engage in specific tasks. In recent years, it has been reported that IBS and chronic pain belong to the central sensitization syndromes (Ford et al., 2020). Neuroimaging research has provided evidence that brain-gut interactions occur in functional gastrointestinal disease (Mayer et al., 2006). Furthermore, previous studies have revealed that there is a link between pain modulation and altered brain networks, including salience network (SN), the default mode network (DMN), and executive control network in IBS. Evidence has shown that these changes in brain networks can affect pain modulation, cognitive, self-awareness, sensorimotor, and emotional aspects of IBS (Liu et al., 2016; Qi et al., 2016).

Several fMRI studies investigating the effect of acupuncture on IBS based on amplitude of low-frequency fluctuations, and functional connectivity methods have demonstrated widespread structural and functional alterations in regional brain activity in IBS-D patients (Ma et al., 2020; Geng et al., 2021; Liu et al., 2021). Furthermore, alterations in regional brain activity of IBS-D patients before and after acupuncture are correlated with changes in IBS symptom severity score (IBS-SSS) and quality of life (Ma et al., 2020; Liu et al., 2021). As we all know, the brain is a complex system with multiple functional brain networks. Moreover, brain regions and networks do not function independently (Liao et al., 2020). However, previous research has mainly focused on few *priori* brain networks and little attention has been paid to the interaction between brain networks. Therefore, exploring the functional connectivity within one specific brain region in acupuncture treatment for IBS may not be sufficient. To date, it remains largely unknown where and how specific alterations in brain inter-network alleviate symptoms in IBS-D patients after acupuncture treatment.

Therefore, in this study, alterations in inter-network functional connectivity in IBS-D patients with acupuncture treatment were explored. First, using an independent component analysis (ICA) approach, the different functional connectivity of inter-network between IBS-D patients and healthy controls (HCs) was determined. Second, the altered functional connectivity of inter-network in patients with IBS-D before and after acupuncture treatment was explored. Furthermore, the relationship between alterations in neural activity and clinical outcomes in patients with IBS-D was also explored. It was hypothesized that inter-network functional connectivity was different between IBS-D patients and HCs. Moreover, altered inter-network functional connectivity in IBS-D patients before and after acupuncture treatment was associated with changes in clinical outcomes.

## Materials and Methods

### Participants

A total of 74 IBS-D and 31 HCs were recruited through advertisement (Table 1). The study was approved by the ethic institutional review board of Jiangsu Province Hospital of Chinese Medicine, and written informed consent was signed by all participants. The inclusion criteria for patients with IBS-D were (1) Rome IV diagnostic criteria; (2) aged between 18 and 70 years old; (3) right handiness; (4) the baseline of IBS-SSS ≥ 75; (5) no drug intervention at least 2 weeks and no acupuncture intervention for 3 months before enrollment; (6) no participation in other studies during the same period; (7) no obvious abnormality of colonoscopy and biochemical examination; and (8) no claustrophobia. The exclusion criteria for patients with IBS-D were (1) intestinal organic lesions, or systemic diseases affecting gastrointestinal motility; (2) an abdominal or rectum and anus operation history; (3) no other organic disorder; (4) in pregnancy, lactation, postpartum ≤ 12 months and susceptible to allergies; (5) other treatments at the same time; (6) a neurology psychiatric disorder history; (7) metal allergy or severe needle fear. The inclusion criteria for HCs were (1) right handiness; (2) no neurology psychiatric disorder history; (3) gender and age-matched with IBS-D patients; (4) no other organic or functional disorder.

**TABLE 1:** The demographic and clinical characteristics in IBS-D/HCs.

	**IBS-D (*n* = 74)**	**HCs (*n* = 31)**	***P***
Sex	37/27	15/16	0.33
Age	42.48 ± 11.60	38.93 ± 12.86	0.20

*IBS-D, Diarrhea-Predominant Irritable Bowel Syndrome; HCs, Healthy Controls.*

### Outcome Measures

The acupuncture treatment period for patients with IBS-D was 6 weeks and included a 2-baseline week period. All the IBS-D participants were required to complete the fMRI examination before and after acupuncture treatment, and HCs only performed fMRI at baseline. In addition, the IBS-SSS and Hamilton Anxiety Scale (HAMA) were estimated as secondary outcomes to access the symptom severity and psychologic factors in IBS-D patients, respectively. All outcomes were measured before and after acupuncture treatment.

### Interventions

Diarrhea-predominant irritable bowel syndrome patients received acupuncture treatment for 6 weeks, which has previously been reported to be effective for IBS-D patients (Ma et al., 2020; Pei et al., 2020). Acupuncture at Baihui (GV20), Yintang (GV29), Taichong (LR3), Zusanli (ST36), Sanyinjiao (SP6), Tianshu (ST25), and Shangjuxu (ST37) acupoints were conducted by the same professional acupuncturist with more than 2 years of clinical experience. Disposable sterile needles (30 mm × 40 mm, Hwato, Suzhou, China) were used for acupuncture treatment. A tincture of iodine and alcohol was used to clean the skin, and the acupuncture needles were inserted into SP6, ST36, and ST37 (25 mm), ST25 (25 to 40 mm), LR3 (15 mm), GV20, and GV29 (15 mm). The twirling, lifting, and thrusting were manipulated once every 10 min, for three times, to obtain deqi sense with soreness, numbness, heaviness, and distention. Acupuncture treatment was administered once every other day, three times a week, 30 min each time, for a total of 18 times. The HCs did not receive any acupuncture treatment.

### MRI Data Acquisition

Images Data were obtained using a 3.0-T Siemens scanner with an 18-channel phased-array head coil at the radiology department, Jiangsu Province Hospital of Chinese Medicine, Nanjing, Jiangsu, China. All IBS-D patients underwent fMRI examination before and after acupuncture treatment, while HCs were screened only at baseline. Earplugs were used to reduce the scanner noise, and the patient’s head was fixed using foam pads. The imaging parameters between IBS-D patients and their matched HCs were similar. Echo-planar imaging (EPI) sequence was used to obtain fMRI images using the following parameters: repetition time (TR) 2310 ms, echo time (TE) 21 ms, flip angle 90°, 42 axial slices for each data volume, a field of view (FOV) 224 mm × 224 mm, matrix size 64 × 64, volume numbers 210, voxel size 3.5 × 3.5 × 3.5 mm, slice gap = 0 mm, and slice thickness 3.5 mm. Additionally, a T1-weighted image was collected using a sagittal three-dimensional magnetization-prepared rapid gradient echo with the following parameters: TR/TE = 2300 ms/2.19 ms, FOV = 256 mm × 256 mm, voxel size = 1.0 × 1.0 × 1.0 mm, matrix size: 256 × 256, flip angle = 9°, slice gap = 0 mm, slice thickness = 1 mm and 176 slices. All participants were instructed to close their eyes, relax, respire normally, move and think as little as possible, and all subjects were confirmed not to have fallen asleep during imaging.

### fMRI Data Preprocessing

Resting-state BOLD data preprocessing was performed using Data Processing & Analysis for Brain Imaging (DPABI^1^) (Yan and Zhang, 2010; Yan et al., 2016), which combined SPM12^2^. The main processing steps were as follows: (1) discarding the first 10 volumes; (2) slice timing (The scanning sequence started from the odd-numbered layers, and the number of scanning layers was 42) and head motion realignment (data > 3 mm of the maximal translation or 3° of the maximal rotation were removed); (3) spatial normalization and smoothing with a Gaussian kernel of 6 mm × 6 mm × 6 mm full-width at half maximum (Ashburner, 2007); (4) temporal filtering (0.01–0.08 Hz), and linear trend removal; (5) regressing of Friston-24 motion parameters, white matter, and cerebrospinal fluid signals (Friston, 2015; Murphy and Fox, 2016).

### Independent Component Analysis

**FIGURE 1:**
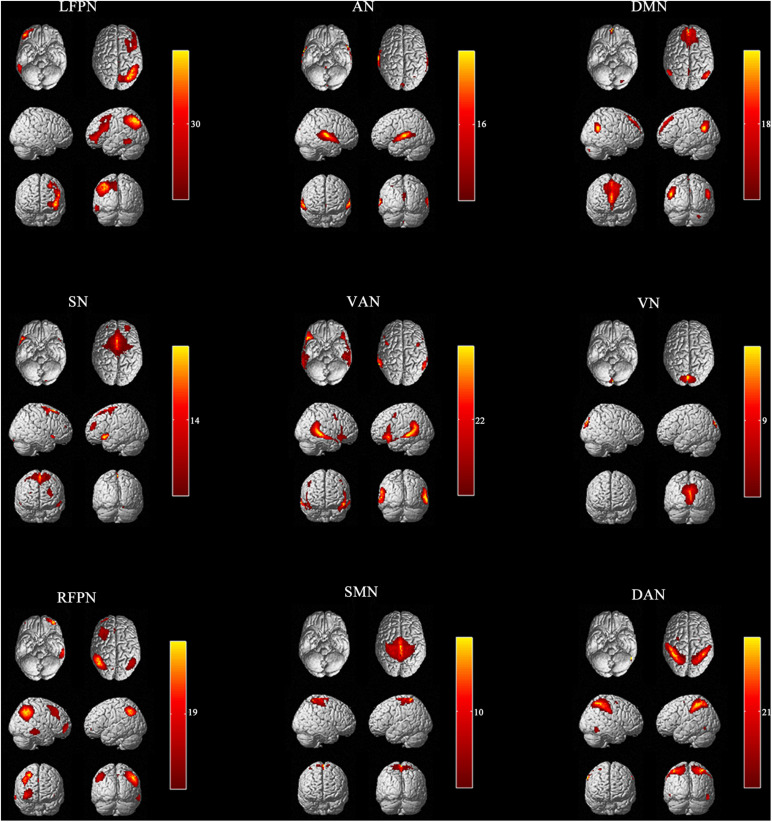
Nine independent components in spatial maps. LFPN, left frontoparietal Network; DMN, anterior default mode network; SN, salience network; VAN, ventral attention network; AN, auditory network; VN, visual network; SMN, sensorimotor network; DAN, dorsal attention network; RFPN, right frontoparietal network; L, left; R, right.

Independent component analysis is data-driven and provides information about functional connectivity on a whole-brain scale (van den Heuvel and Hulshoff Pol, 2010). ICA was used to parcellate fMRI data using the GIFT toolbox^3^ (Wang et al., 2020). A total of 36 independent components were generated. Finally, 9 functional networks were identified as independent components of peak activations in gray matter with low spatial overlap and primarily low-frequency power of vascular, ventricular, motion, and susceptibility artifacts (Figure 1). The Left Frontoparietal Network (LFPN), Default Mode Network (DMN), Salience Network (SN), Ventral Attention Network (VAN), Auditory Network (AN), Visual Network (VN), Sensorimotor Network (SMN), Dorsal Attention Network (DAN), and Right Frontoparietal Network (RFPN) were retrieved from all participants.

### Inter-Network Functional Connectivity Analysis

**FIGURE 2:**
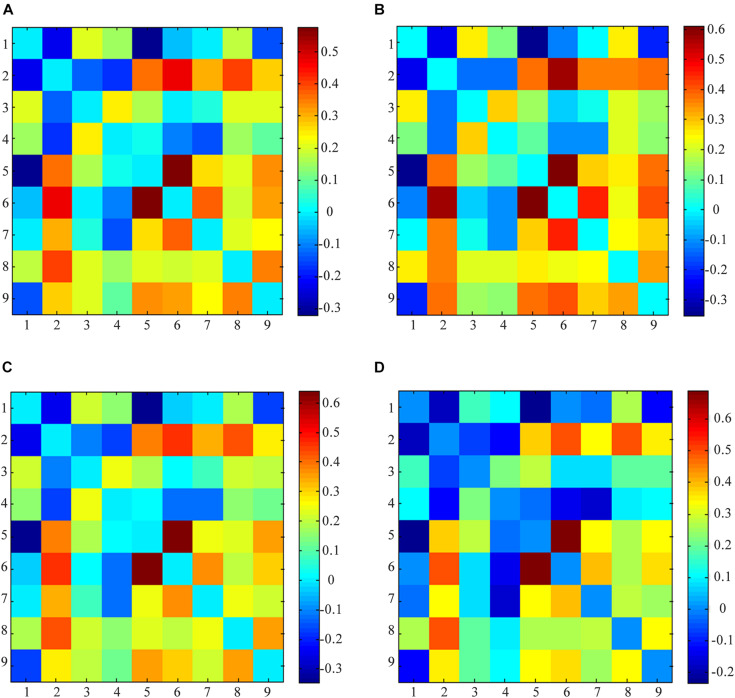
Averaged functional networks activity. **(A)** Averaged functional networks of all subjects (between IBS-D before acupuncture and HCs); **(B)** Averaged functional networks of HCs; **(C)** Averaged functional networks of IBS-D patients before acupuncture; **(D)** Averaged functional networks of IBS-D patients after acupuncture; (1) anterior default mode network (DMN); (2) auditory network (AN); (3) left frontoparietal Network (LFPN); (4) right frontoparietal network (RFPN); (5) dorsal attention network (DAN); (6) sensorimotor network (SMN); (7) visual network (VN); (8) ventral attention network (VAN); (9) salience network (SN).

Linear de-trending, de-spiking, and low-pass filtering were conducted for all participants for the functional networks of interest before calculating the internetwork functional connectivity (Buckner et al., 2008; Luo et al., 2014; Liu et al., 2015). The Pearson’s correlation coefficients for each functional network pair in each participant were obtained. Finally, to improve the normality, a symmetric 9 × 9 correlation matrix was constructed using Fisher’s z-transformation (Figure 2). A false discovery rate was employed to correct for multiple comparisons, and *P* < 0.05 was considered to be statistically significant.

### Statistical Analysis

The demographic characteristics and clinical outcomes were analyzed using SPSS software 26.0 (IBM, United States). Dichotomous variables were analyzed using the chi-square test whereas continuous variables were compared using two-dependent *t*-tests. Changes in clinical outcomes after acupuncture treatment in IBS-D patients were analyzed using the paired *t*-test. The statistical significance threshold was set at *P* < 0.05. The fMRI data was compared between IBS and HCs using a two-sample *t*-test, and changes in IBS before and after acupuncture treatment were analyzed using the paired *t*-test (*P* < 0.05, FDR corrected). Pearson correlation analyses were performed to determine the association between changes in IBS-SSS, HAMA, and changes in the inter-network functional connectivity in IBS-D patients.

## Results

### Demographic and Clinical Characteristics

The demographic and clinical characteristics of the two groups are presented in Table 1. There were no significant differences in age and sex between IBS-D patients and HCs (*P* > 0.05; Table 1). After acupuncture, the IBS-SSS (the degree of abdominal pain, abdominal pain days, the degree of abdominal distension, satisfaction with bowel movements, life influence degree) and HAMA decreased in IBS-D patients (*P* < 0.05; Table 2).

**TABLE 2:** The clinical outcomes of IBS-D patients before and after acupuncture treatment (Mean ± SD).

**Item**	**Before acupuncture**	**After acupuncture**	**t**	***P***
IBS-SSS	250.26 ± 88.84	121.95 ± 62.88	9.15	0.00
The degree of abdominal pain	42.82 ± 27.16	18.85 ± 17.82	5.98	0.00
Abdominal pain days	45.90 ± 33.30	19.49 ± 18.77	4.25	0.00
The degree of abdominal distension	26.92 ± 26.70	9.23 ± 13.35	4.65	0.00
Satisfaction with bowel movements	67.95 ± 20.38	37.41 ± 18.22	7.76	0.00
Life influence degree	65.00 ± 23.51	36.72 ± 19.43	7.26	0.00
HAMA	17.69 ± 8.76	10.88 ± 5.75	4.62	0.00

*IBS-D, Diarrhea-Predominant Irritable Bowel Syndrome; Irritable Bowel Syndrome Symptom Severity Score (IBS-SSS); HAMA, Hamilton Anxiety Scale.*

### Functional Connection Matrix Analysis

**FIGURE 3:**
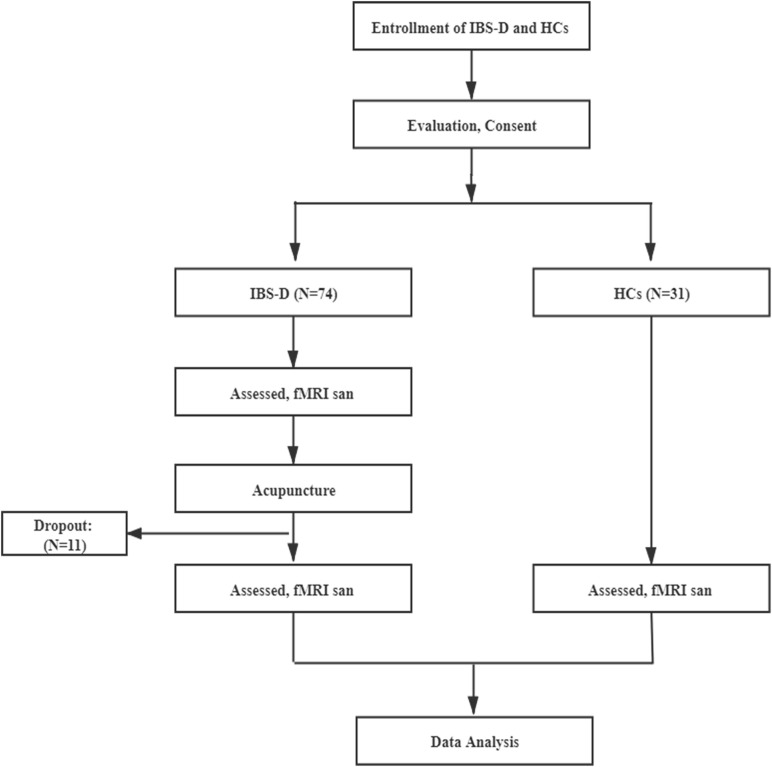
Procedures and data used for the study.

In the comparation between IBS-D before acupuncture and HCs, ten IBS-D patients (eight with head movement, two with parameter differences) and two HCs (head movement) were excluded (Figure 3). Finally, the fMRI data of 64 IBS-D patients before acupuncture were compared with 29 HCs. Eleven IBS-D patients dropped out during acupuncture treatment, Eleven IBS-D were received sham acupuncture. The fMRI of 42 IBS-D patients with completed true acupuncture treatment were analyzed.

**FIGURE 4:**
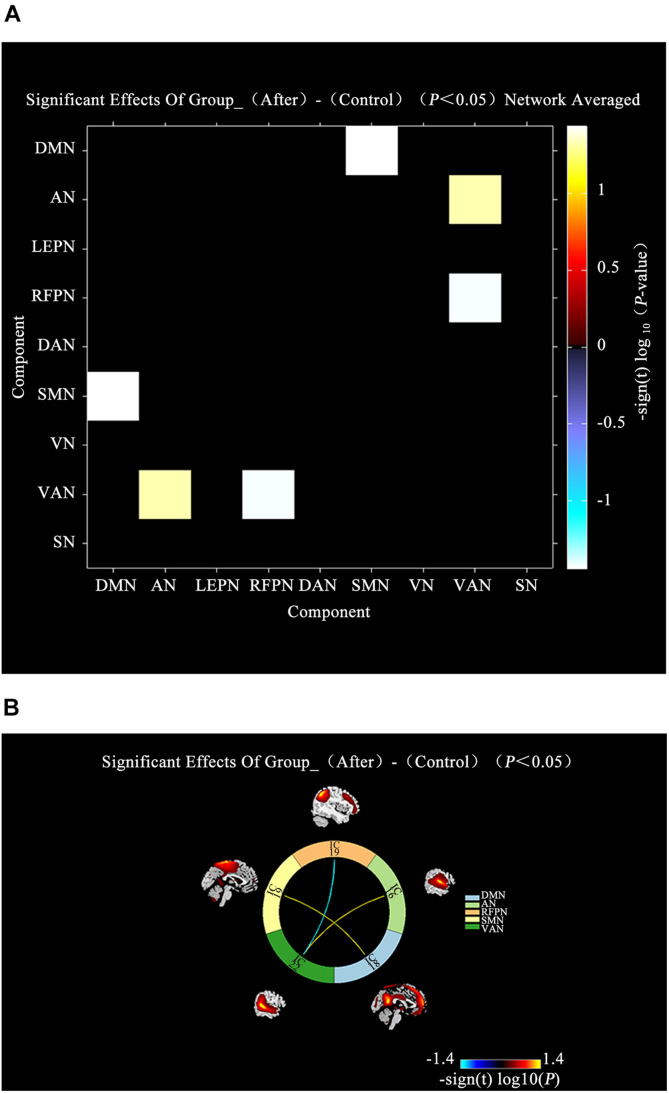
Connectivity comparisons of large-scale network between the IBS-D patients with HCs. **(A)** Connectivity matrix of functional network connectivity of large-scale network in IBS-D patients after acupuncture and HCs. **(B)** Connectivity of functional network connectivity of large-scale network in IBS-D patients after acupuncture and HCs; Hot colors represent positive functional network connectivity, and cool colors represent negative functional connectivity. LFPN, left frontoparietal Network; DMN, anterior default mode network; SN, salience network; VAN, ventral attention network; AN, auditory network; VN, visual network; SMN, sensorimotor network; DAN, dorsal attention network; RFPN, right frontoparietal network; L, left; R, right.

**FIGURE 5:**
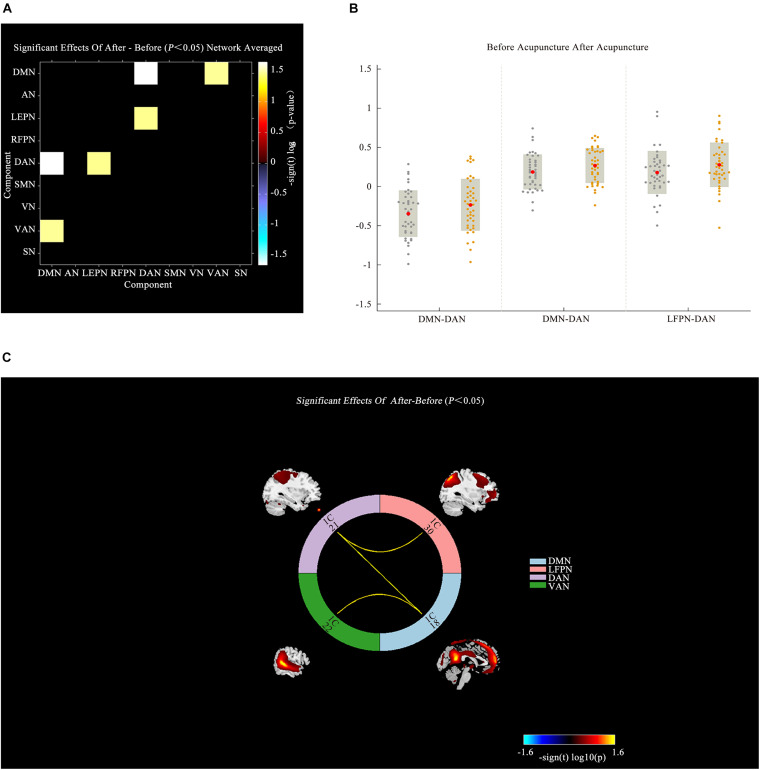
Connectivity comparisons of large-scale network between the IBS-D patients before and after acupuncture. **(A)** Connectivity matrix of functional network connectivity of large-scale network in IBS-D before and after acupuncture. **(B)** The changes of functional network connectivity of large-scale network in IBS-D before and after acupuncture. **(C)** Connectivity of functional network connectivity of large-scale network in IBS-D patients before and after acupuncture; Hot colors represent positive functional connectivity, and cool colors represent negative functional connectivity. LFPN, left frontoparietal Network; DMN, anterior default mode network; SN, salience network; VAN, ventral attention network; AN, auditory network; VN, visual network; SMN, sensorimotor network; DAN, dorsal attention network; RFPN, right frontoparietal network; L, left; R, right.

A comparison between IBS-D patients and HCs showed increased AN-VAN and SMN-DMN and decreased RFPN-VAN (*P* < 0.05; Figure 4) after acupuncture. Interestingly, there was no significant difference in functional connectivity between IBS-D patients before acupuncture and HCs (*P* > 0.05). However, as illustrated in Figure 5, increased functional connectivity in IBS-D patients was reported after acupuncture treatment, including DMN-DAN, DAN-LFPN, and DMN-VAN (*P* < 0.05).

### Correlation Analysis of Altered Inter-Network Functional Connectivity and Clinical Information

**FIGURE 6:**
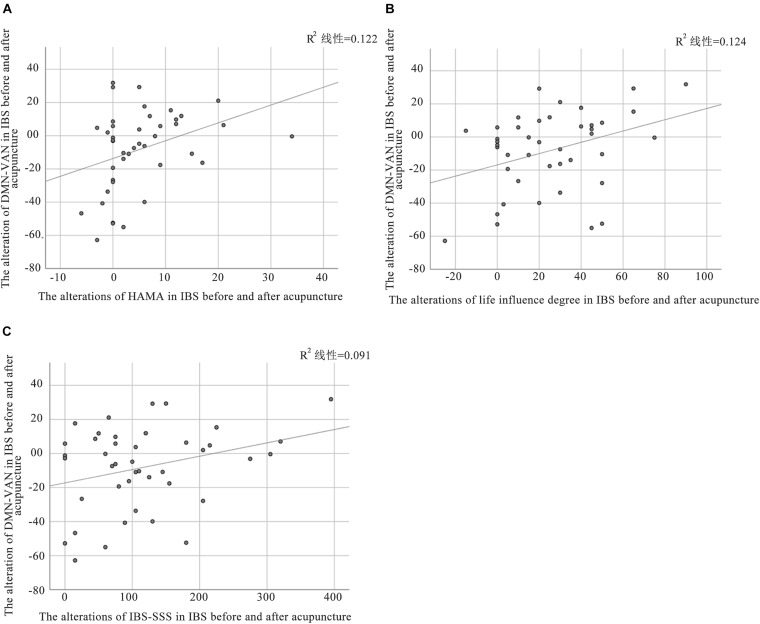
Correlation between altered FC and clinical outcomes in patients with IBS. **(A)** The alteration of DMN-VAN was positively correlated with the changes in HAMA (*P* = 0.024, r = 0.35); **(B)** The alteration of DMN-VAN was positively correlated with the changes in life influence degree (*P* = 0.022, r = 0.32); **(C)** The alteration of DMN-VAN was were positively correlated with the changes in IBS-SSS (*P* = 0.05, r = 0.30).

In this study, a relationship between alteration of functional connectivity and alterations of clinical outcomes was found. Pearson correlation analysis showed that the alteration of DMN-VAN was positively correlated with changes in HAMA (*P* = 0.024, r = 0.35) and life influence degree (*P* = 0.022, r = 0.32). While the alteration of DMN-VAN was positively correlated with changes in IBS-SSS at the boundary of significance threshold (*P* = 0.05, r = 0.30). However, there was no significant correlation between other alterations in the inter-network functional connectivity and clinical variables in IBS-D patients (Figure 6).

## Discussion

This study for the first time, systematically investigated functional connectivity in IBS-D patients within large-scale networks before and after acupuncture treatment. Compared with other data processing methods (seed-based or graph-based), ICA is less sensitive to confound physiologic noise and head motion (Calhoun and Adali, 2012). Therefore, the ICA method was used to analyze functional networks in all participants. The functional connectivity matrices of HCs and IBS-D patients before and after acupuncture treatment were separately constructed. First, the effect of acupuncture on ameliorating abdominal pain, abdominal distension, satisfaction with bowel movements, life influence degree, and anxiety (IBS-SSS, HAMA) was verified (*P* < 0.05). Subsequently, the potential inter-network mechanisms underlying acupuncture treatment for IBS-D patients were investigated. The functional connectivity between SMN and DMN, AN and VAN, and RFPN and VAN in IBS-D patients after acupuncture was found to be different from HCs and was speculated to be related to pathogenesis in IBS-D patients (*P* < 0.05). Interestingly, there was no significant difference in functional connectivity between IBS-D patients before acupuncture treatment and HCs (*P* > 0.05). Furthermore, the functional connectivity associated with DMN-DAN, DAN-LFPN, and DMN-VAN showed increased changes in IBS-D patients after acupuncture treatment, which might reveal a potential regulatory mechanism of acupuncture’s effect on IBS-D patients (*P* < 0.05). Pearson correlation analysis between the alterations of functional connectivity and clinical outcomes revealed that the alteration of DMN-VAN was positively correlated with changes in HAMA, life influence degree, and IBS-SSS. These results also revealed the effect of acupuncture stimulation at Baihui (GV20), Yintang (GV29), Taichong (LR3), Zusanli (ST36), Sanyinjiao (SP6), Tianshu (ST25), and Shangjuxu (ST37) on some large-scale brain networks of IBS-D patients, and might suggest a potential mechanism of acupuncture’s effect on IBS-D.

The effect and safety of these acupuncture acupoints for IBS were reported in our previous clinical research. After 6 weeks of acupuncture intervention, the total IBS-SSS decreased by 123.51, while the IBS-quality of life total score increased by 13.35 in IBS patients (Pei et al., 2020). Moreover, the number of pain days, defecation satisfaction, life disturbance degree, and total score of the IBS patients group were lower than polyethylene glycol or pinaverium bromide (Li et al., 2017). Brain-gut axis interactions have a close relationship with the regulation of digestion (Mayer and Saper, 2000). Recent research on the mechanism of acupuncture therapy shows that altered brain-gut axis plays a crucial role in the pathophysiological changes involved in IBS (abdominal hypersensitivity) (Xu et al., 2018). Several studies have shown that acupuncture stimulation can regulate the gut microbiota in IBS. A recent animal study showed that electroacupuncture reduced Simpson diversity index, Shannon diversity index, and Fusobacterium, as well as the mRNA and protein levels of IL-18 (Song et al., 2020). Our previous clinical research reported that Firmicutes and Simpson’s index were reduced, while Bacteroidetes, Shannon index, Proteobacteria, and short-chain fatty acid were increased in IBS-D patients after acupuncture treatment. The results demonstrated that the effect of acupuncture may be related to the regulation of the structure and diversity of the gut microbiota and fecal short-chain fatty acids (Chen L. et al., 2021).

In our previous studies, we investigated the fractional amplitude of low-frequency fluctuation values, functional connectivity, degree, clustering coefficient, and local efficiency in IBS-D patients using the same acupoints reported in this study. Compared with the pre-treatment condition, the amplitude of low-frequency fluctuation values in the right postcentral gyrus, left precentral gyrus, right supplementary motor area, and right superior frontal gyrus was found to have increased (Liu et al., 2021). A previous study evaluated functional connectivity using bilateral hippocampus regions, and the results showed that brain activity differed in the hippocampus, insula, temporo-sphenoid lobe, and occipital lobe in IBS-D patients with acupuncture treatment (Geng et al., 2021). Additionally, increased degrees in the right middle occipital gyrus, and the clustering coefficients and local efficiency in the left superior occipital gyrus were also revealed in IBS-D patients with acupuncture treatment (Ma et al., 2020).

Research has shown that the DMN network plays a pivotal role in processes that occur in the presence of pain (Zou et al., 2021). This study results were consistent with previous studies reporting different DMN in IBS patients after acupuncture treatment. Hall et al. (2010) reported increased DMN connectivity with pain- and emotion-associated brain regions, while Qi et al. (2016), reported that IBS patients showed decreased DMN inter-regional functional connectivity between the anterior cingulate cortex and precuneus, the medial orbital of the superior frontal gyrus and precuneus, and the middle temporal gyrus and precuneus (Qi et al., 2016). The present study showed that changes in DMN in IBS-D patients caused by acupuncture stimulation were consistent with DMN which influences specific brain networks with functional connectivity following acupuncture treatment in other functional gastrointestinal diseases. Sun et al. found that acupuncture treatment with deqi in functional dyspepsia mortified the abnormal functional connectivity within the salience network, and participated in the adaptive modulation of disrupting the relationship between the SN and DMN.

As a major ascending pathway of pain, SMN play a role in the integration of sensory, cognitive, and affective functions (Craggs et al., 2007), and is also involved in the neuro-regulation of IBS (Icenhour et al., 2017). The posterior insula, a primary interoceptive cortex, represents interoceptive processing, and it is considered an important multimodal convergence region for sensorimotor, such as viscera and pain (Chang et al., 2013; Zhao et al., 2018). Similarly, SMA is a motor brain region that participates in the integration of affective and cognitive functions (Nachev et al., 2008). Yan et al. found that the depressed patients have an abnormal functional integration of SMA, which contributes to psychomotor retardation (Yan et al., 2019; Sahib et al., 2020). A recent study showed that abnormal functional connectivity in IBS patients with depressive symptoms is related to insula and SMA (Li et al., 2021). Accumulating evidence suggests that enhanced functional connectivity of insula within the SMN may reflect increased ascending input and processing of visceral sensitivity in IBS patients with visceral hypersensitivity (Icenhour et al., 2017). Therefore, the present study finding was consistent with previous research, where the abnormal brain network SMN is a factor that may lead to IBS.

The DAN consists of regions in the frontal (frontal eye field) and posterior parietal (intraparietal sulcus, superior parietal lobe) cortex (Corbetta and Shulman, 2002). It is reported that DAN is activated when attention is oriented in space, including maintaining spatial priority maps, saccade planning, and visual working memory (Jerde et al., 2012). VAN is associated with the orientation of perceptual dimension, and its main brain regions are the ventrolateral prefrontal cortex and bilateral temporal-parietal junction (Corbetta et al., 2008). Recently, the clustering coefficient and small worldness of DAN and VAN in major depressive disorders were found to have increased significantly (Chen et al., 2019). Considering IBS symptoms, a complex interplay among visceral sensation regulation, pain processing, and anxiety and depression, which are associated with higher incidence is reported (Fond et al., 2014). Therefore, in this study, the brain activities of DAN and VAN were found to be associated with depression, evidenced by the changes in IBS-D before and after acupuncture treatment. These results indicated that the brain network of DAN and VAN is important in regulating brain activity in IBS.

Prior neuropsychological and neuroimaging studies suggest that RFPN is recognized as an important brain network in cognitive control and top-down modulation in ICA (Corbetta and Shulman, 2002; Crone et al., 2006). Previous studies have revealed that key regions of the RFPN include the ventrolateral prefrontal cortex (PFC), dorsolateral PFC, and the parietal gyrus. These brain regions have direct or indirect connections with cognitive control of pain, somesthesis, perception, affective (anterior cingulate cortex, medial PFC, and amygdala), and sensory (primary somatosensory cortex (SI) and S2/insula) pain process components (Wiech et al., 2008; Smith et al., 2009; Bushnell et al., 2013). Moreover, girls with IBS exhibit decreased gray matter volume in the dorsolateral PFC (Bhatt et al., 2019). The finding in this study is consisted with previous reports. In this study, we found that RFPN and VAN between IBS and HCs, and DAN and LFPN in IBS before and after acupuncture treatment were different. We thus speculate that changes in FC of RFPN in IBS patients may reflect the brain’s self-compensatory adaptation/coping reaction to continued influence of IBS (Damme et al., 2008; Mansour et al., 2014). Evidence from previous studies shows that acupuncture treatment has non-specific effects on chronic pain (Kong et al., 2009; Peihong et al., 2021). Therefore, the present results indicate that acupuncture may alleviate abdominal pain by altering the self-compensatory adaptation/coping process.

Auditory network is the most affected brain network during aberrant objective sound perception (Crippa et al., 2010). It has long been described in terms of core, belt, and parabelt regions (Kaas and Hackett, 2000), and the posteromedial two-thirds of Heschl’s Gyrus is composed of the core auditory cortex (Nourski et al., 2014). The temporal lobe has an important influence on mental activity and perceptual function, which consists of the superior temporal gyrus, middle temporal gyrus, and inferior temporal gyrus (Behrmann et al., 2016). Part of the superior temporal gyrus is occulted into the lateral fissure and it is terms as transverse temporal gyrus. Therefore, we inferred that the altered brain activity of the core AN local that Heschl’s Gyrus may be related to affective in IBS. Ma et al. reported that ALFF values were decreased in the right superior temporal gyrus of patients with IBS (Ma et al., 2015). Compared with HCs, the functional connectivity between the left hippocampus and left superior temporal gyrus increased, while that between the right hippocampus and left inferior temporal gyrus decreased in IBS-D patients. Additionally, the functional connectivity between the left hippocampus and right inferior temporal gyrus was enhanced in IBS-D patients, while in the right hippocampus, left inferior temporal gyrus and left superior temporal gyrus were increased in IBS patients after acupuncture treatment (Geng et al., 2021). These results were consistent with previous findings, indicating that the role of AN in IBS is important.

Unexpectedly, there was no difference of network functional connectivity between IBS and HCs before acupuncture treatment. Moreover, we searched the PubMed and did not find any comparison of inter-network functional connectivity between IBS and HCs. Further research is advocated to explore interactions in the inter-network functional connectivity in IBS-D.

### Limitations

The current study has some limitations. First, the number of subjects was relatively small. Second, we did not include a sham acupuncture group, thus could not determine whether the alterations in functional connectivity were associated with the acupoint specificity. Third, IBS-D is a chronic disease, the onset of symptoms is not the same as the period of improvement, and the brain functional connectivity may differ at different periods. However, there were no time restrictions when conducting fMRI examinations. Therefore, future research with a much larger sample, sham acupuncture, and uniform fMRI examination periods are warranted to elucidate the progressive functional connectivity alterations in IBS-D patients.

## Conclusion

This study demonstrates the feasibility and reliability of acupuncture in the treatment of IBS-D. The acupuncture points used in this study can also be used to treat IBS as a complementary and alternative treatment without side effects. Using the ICA method, we show that alterations in DMN-DAN, DAN-LFPN, and DMN-VAN may be considered when interpreting the therapeutic effects of acupuncture at Baihui (GV20), Yintang (GV29), Taichong (LR3), Zusanli (ST36), Sanyinjiao (SP6), Tianshu (ST25), and Shangjuxu (ST37) in IBS-D. Therefore, this study lays the foundation for future investigations into the mechanisms by which acupuncture affects large-scale brain functional inter-network in IBS-D. Specifically, the present study shows that inter-network functional connection is involved in several important hubs in the large-scale networks, and can be considered important biomarkers in IBS-D patients.

## Data Availability Statement

The original contributions presented in the study are included in the article/supplementary material, further inquiries can be directed to the corresponding author/s.

## Ethics Statement

The studies involving human participants were reviewed and approved by Ethics Committee of the Jiangsu Province Hospital of Chinese Medicine. The patients/participants provided their written informed consent to participate in this study.

## Author Contributions

LP and LC: conception and design. HN: data collection. YS: data analysis. TZ: writing. JZ: data curation and supervision. JG: english-language revision. ZM and JS: revision. All authors contributed to the final version of the manuscript and approved the submitted version.

## Conflict of Interest

The authors declare that the research was conducted in the absence of any commercial or financial relationships that could be construed as a potential conflict of interest.

## Publisher’s Note

All claims expressed in this article are solely those of the authors and do not necessarily represent those of their affiliated organizations, or those of the publisher, the editors and the reviewers. Any product that may be evaluated in this article, or claim that may be made by its manufacturer, is not guaranteed or endorsed by the publisher.

## Footnotes

1
http://rfmri.org/dpabi

2
http://www.fil.ion.ucl.ac.uk/spm

3
http://mialab.mrn.org/software/gift/
